# Nylon mesh-based sample holder for fixed-target serial femtosecond crystallography

**DOI:** 10.1038/s41598-019-43485-z

**Published:** 2019-05-06

**Authors:** Donghyeon Lee, Sangwon Baek, Jaehyun Park, Keondo Lee, Jangwoo Kim, Sang Jae Lee, Wan Kyun Chung, Jong-Lam Lee, Yunje Cho, Ki Hyun Nam

**Affiliations:** 1 Department of Mechanical Engineering, POSTECH, Pohang, 37673 Republic of Korea; 2 Department of Materials Science and Engineering, POSTECH, Pohang, 37673 Republic of Korea; 3 Pohang Accelerator Laboratory, POSTECH, Pohang, 37673 Republic of Korea; 4 Department of Life Science, POSTECH, Pohang, 37673 Republic of Korea; 5 Division of Biotechnology, Korea University, Seoul, 02841 Republic of Korea; 6 Institute of Life Science and Natural Resources, Korea University, Seoul, 02841 Republic of Korea

**Keywords:** X-ray crystallography, Nanocrystallography

## Abstract

Fixed-target serial femtosecond crystallography (FT-SFX) was an important advance in crystallography by dramatically reducing sample consumption, while maintaining the benefits of SFX for obtaining crystal structures at room temperature without radiation damage. Despite a number of advantages, preparation of a sample holder for the sample delivery in FT-SFX with the use of many crystals in a single mount at ambient temperature is challenging as it can be complicated and costly, and thus, development of an efficient sample holder is essential. In this study, we introduced a nylon mesh-based sample holder enclosed by a polyimide film. This sample holder can be rapidly manufactured using a commercially available nylon mesh with pores of a desired size at a low cost without challenging technology. Furthermore, this simple device is highly efficient in data acquisition. We performed FT-SFX using a nylon mesh-based sample holder and collected over 130,000 images on a single sample holder using a 30 Hz X-ray pulse for 1.2 h. We determined the crystal structures of lysozyme and glucose isomerase using the nylon mesh at 1.65 and 1.75 Å, respectively. The nylon mesh exposed to X-rays produced very low levels of background scattering at 3.75 and 4.30 Å, which are negligible for data analysis. Our method provides a simple and rapid but highly efficient way to deliver samples for FT-SFX.

## Introduction

Serial femtosecond crystallography (SFX) using an X-ray free electron laser (XFEL) can be used to visualize the crystal structures of biomolecules at room temperature without radiation damage, a concept known as diffraction before destruction^1^. This technique provides biologically more accurate structural information than conventional synchrotron-based X-ray crystallography. Since crystal samples exposed to the XFEL exhibit radiation damage immediately after diffraction, they must be serially delivered to the X-ray interaction point to collect the full diffraction dataset^2^. To continuously deliver a large number of crystal samples, various sample delivery systems, such as liquid jet injectors using the gas dynamic virtual nozzle^3^, electrospinning^4^, LCP (lipidic cubic phase) injectors^5^, acoustic injectors^6^, or viscous media^7–10^, have been developed. Fixed-target serial femtosecond crystallography (FT-SFX) can be advantageous over other methods because it delivers crystals stably and can dramatically reduce sample consumption with a high hit rate^11–27^. In addition, while sample delivery using a common injector involves physical impact on the crystal sample, crystal size filtering, and pressure during loading, the fixed-target method avoids such physical damage during crystal preparation^28^. FT-SFX experiments may be performed at both cryogenic and ambient temperatures^28^. At room temperature, crystals in the sample holder may get dehydrated during data collection^14,29,30^. This may be overcome by using strategies to maintain the humidity of the surroundings^28^, such as continuous flow of humidified gas^14^, or surrounding in a viscous grease or oil^20^, or sealing the sample holder with a film^13^. At cryogenic temperature, crystal sample can be stored more straightforwardly by flash-freezing^14,31^. However, this temperature provides a less realistic conformational flexibility for protein function than room temperature^32^.

The fixed-target method can be divided into two approaches, one using a multi-shot goniometer approach using larger crystals, and the other using a multiple microcrystal approach^28^. The multi-shot goniometer method exposes X-rays to renew volumes each time on a large crystal sample^28^. In this strategy, raster, helical, oscillation, and grid data collection methods have been applied for FT-SFX^17,28^. Among them, combination of translation and rotation of crystals, using a goniometer, has been considered useful for the purpose of collecting room-temperature data or for reducing the dose of X-rays exposed to a crystal sample^28^. For example, when X-rays are incident on a large crystal sample, diffraction data are collected while the crystal rotates by 0.1 to 0.5° using a goniometer^16,17,22,25^. After data collection, the X-ray exposure site in the crystal is translated into renew crystal volumes that were not exposed to X-rays earlier, and hence, the crystal is immediately rotated by the goniometer and diffraction data collected^16,17,22,25^. Collecting data for more than 11° of oscillation helps to resolve indexing ambiguities in initial data processing^17^. Moreover, rotation of crystals during data collection is useful for enhancing the data completeness, when the crystal sample is placed in the sample holder with the preferred orientation^17^.

Multiple microcrystal approach refers to a method of collecting diffraction data by exposing X-rays to each crystal sample only once^28^. In order to continuously provide a large number of crystals to the X-ray interaction point in a fixed state, a sample holder capable of supporting many crystals is required. The sample holder should be so designed that the crystal sample can be deposited stably and is uniformly distributed to reduce chances of multi-crystal hit by X-rays^14,19,21,29,31^. Moreover, the material or design should minimize background scattering, generated when X-rays are transmitted through the sample holder^29,31^. Currently, various sample holders, such as silicon nitride^11,18,24^, silicon chips^26^, microfluidic chips^21^, and microgrids^17,19^ are being used for the development of serial crystallography and are successfully implemented in FT-SFX. However, the processes of precise chip fabrication for silicon nitride, silicon chip, and microfluidic chip are challenging and time-consuming^15,21^. In case of micro-grid, it is necessary to change the sample several times while collecting full data since the chip area to accommodate crystals is small^17,19^. Moreover, these sample holders may require suitable mounting holes each time depending on the size and shape of crystals for the stable deposition of crystals. Recently, FT-SFX has been performed by placing a crystal sample between Mylar films without a chip to simplify this process^15^. Nevertheless, it remains to challenge to place the crystal in the holes to prevent the sample-slippage problem. Accordingly, a convenient sample-holding method that resolves the limitation described above should be developed to facilitate experimental access in FT-SFX.

A nylon polymer consists of a polyethylene segment (CH_2_)_n_ separated by a parallel or anti-parallel peptide unit (NH-CO)^33^. These peptide units generate hydrogen bonds between the polymer chains, which imparts the inherent properties of nylon^33^. Among various applications of nylon materials, nylon loops are widely used to mount crystal samples in single crystal X-ray diffraction experiments^34^. This loop provides extremely stable support for crystal samples, and the nylon material produces insignificant X-ray background scattering^34,35^. Owing to this low background scattering, we hypothesized that X-ray-transparent nylon could be used as a sample-holding material for FT-SFX for raster scanning. On the other hand, the larger nylon mesh (pore size: 1.0 mm × 0.9 mm) in the crystal extractor device, used in the hybrid method, has been applied in SFX experiment previously^36^. Here, we introduce a nylon mesh-based sample holder enclosed by a polyimide (as known as Kapton) film for FT-SFX and describe the sample loading method. We performed FT-SFX using the nylon mesh-based sample holder at the Pohang Accelerator Laboratory X-ray Free Electron Laser (PAL-XFEL) and determined the crystal structures of lysozyme and glucose isomerase at 1.65 Å and 1.75 Å, respectively. We analyzed diffuse X-ray scattering from the nylon mesh and discussed the advantages of our method. Our results are applicable not only to FT-SFX but also to serial diffraction data collection using a synchrotron.

## Results and Discussion

### Fabrication scheme for the nylon mesh enclosed by polyimide for crystal mounting

**Figure 1:**
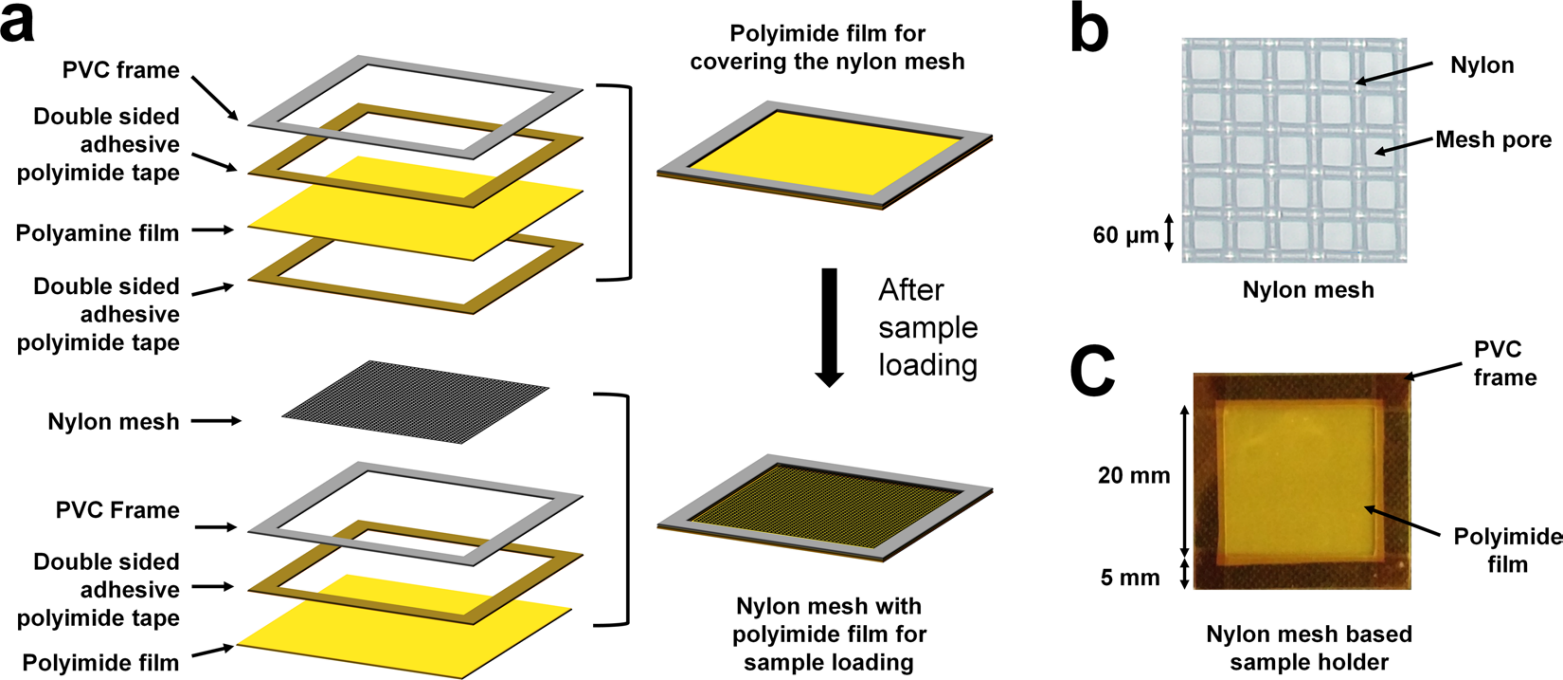
Preparation of the sample holder using the nylon mesh and polyimide film. (**a**) Nylon mesh-based crystal sample holder configuration. (**b**) Close-up view of the nylon mesh used in the FT-SFX experiment. (**c**) Nylon mesh-based sample holder used in FT-SFX experiments.

Nylon generates low-level X-ray scattering and is widely used as a loop material for crystal sample mounting in synchrotron-based X-ray crystallography experiments^11^. When the nylon loop is used as the FT-SFX sample holder, the crystal sample sinks downward by gravity if the holder is set up vertically for X-ray exposure. To overcome this, we used a nylon mesh, and each crystal was seated on a mesh pore to prevent it from sinking. When a crystal sample placed on a mesh is exposed to the atmosphere, the crystallization solution eventually evaporates. This could damage the crystal lattice and reduce the diffraction intensity. Additionally, the salt contained in the crystallization solution may crystallize. In this case, salt crystals exposed to X-rays can generate a strong salt Bragg peak and reduce data quality. Furthermore, dehydration of the crystal sample can change the crystal space group^37^. To prevent the evaporation of the crystal solution, we enclosed the front and back of the nylon mesh in thin polyimide films (Fig. 1a). Accordingly, we fabricated a sample holder based on nylon mesh enclosed by a polyimide film.

In our FT-SFX system, the holder for mounting the sample delivery chip was made of acrylic (Supplementary Fig. S1), which was mounted on a motion stage. The acrylic holder can be equipped with a 30 mm × 30 mm sample holder. We fabricated a nylon mesh-based sample holder of the same size. For easy handling during sample loading and mounting on the acrylic holder, a thin polyimide film (25 μm) to enclose the nylon mesh was surrounded by a 5 mm (width) × 0.3 mm (thickness) PVC frame. The thin polyimide film and PVC frame were attached using 5 mm wide double-sided adhesive polyimide tape (Fig. 1a). The nylon mesh (20 × 20 mm) was placed inside the PVC frame of the polyimide film. Double-sided adhesive polyimide tape was attached between the polyimide films to prevent evaporation of the solution (Fig. 1a). In this experiment, we used a nylon mesh of 20 × 20 mm with 60 μm mesh pores (Fig. 1b). When raster scanning was performed at 50 μm intervals, 400 points were scanned horizontally and vertically. The setup was designed to collect a total of 160,000 diffraction images from a single nylon mesh chip (Fig. 1c). Nylon mesh-based sample holder can be mounted within few seconds on an acrylic mount connected to the stage, which can in turn be accomplished within approximately 3 min. Taken together, the process of mounting the sample holder and preparing the sample for scan occurs very quickly in our system.

### Sample loading

**Figure 2:**
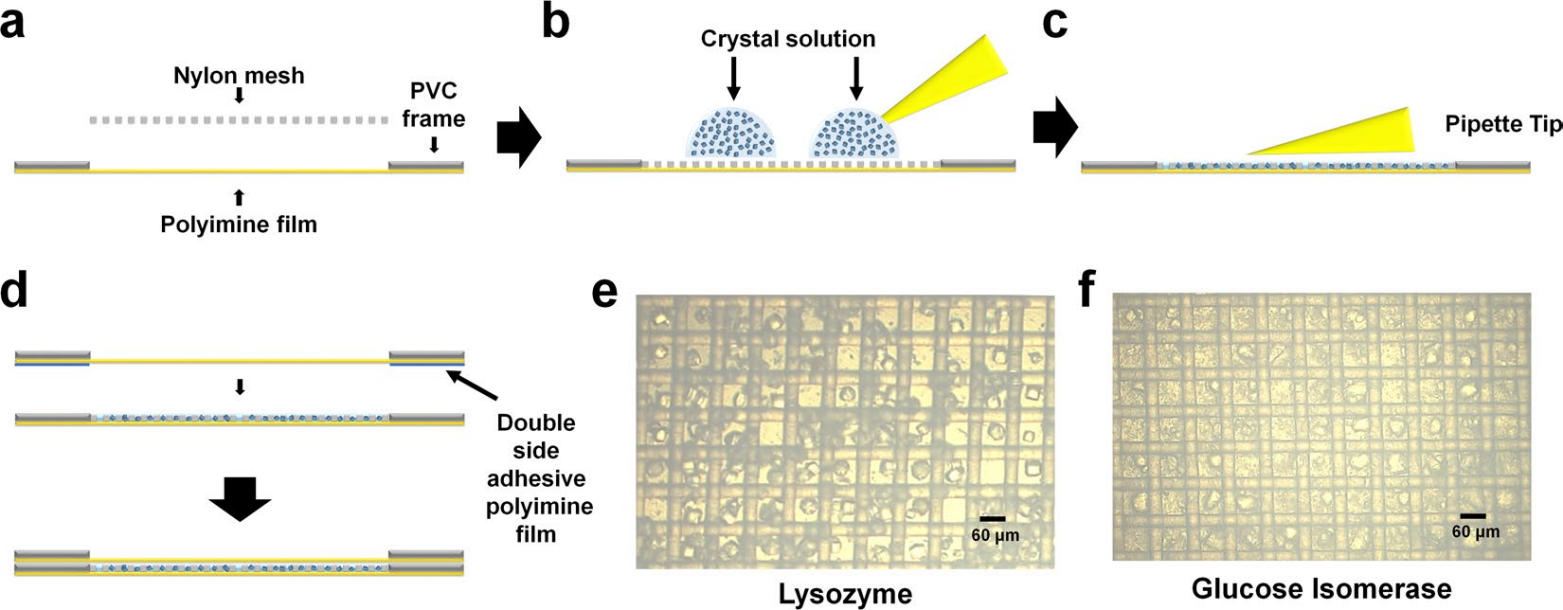
Sample loading on the nylon mesh for FT-SFX. (**a–d**) Scheme of crystal solution loading on the nylon mesh. (**a**) A nylon mesh is placed on a thin polyimide film made of the PVC frame. (**b**) The crystal solution is dispensed onto the nylon mesh using a pipette. (**c**) The crystal solution is spread widely using a pipette tip. (**d**) A thin polyimide film is covered to prevent dehydration of the crystal sample and crystal solution. There is a double-sided adhesive polyimide tape between the two polyimide films. Microscope view of (**e**) lysozyme and (**f**) glucose isomerase on the nylon mesh.

We placed the polyimide film with the PVC frame facing up and placed the nylon mesh inside the PVC frame (Fig. 2a). The protein crystal solution (80 μl) was dispensed on the nylon mesh (Fig. 2b). When the crystal solution was spread evenly using a pipette, the crystallization solution immediately dropped to the bottom of the nylon mesh, and the crystal sample was placed in the mesh hole together with the solution (Fig. 2c). To prevent evaporation of the crystal solution, a nylon mesh with crystals was immediately covered with a polyimide film on the upper layer (Fig. 2d). The crystal solution was sealed between the upper and lower polyimide films using double-sided adhesive polyimide tape to prevent evaporation (Fig. 2d).

To minimize background scattering from the crystal solution, we initially sealed the polyimide film after spreading a 40 μl volume of the solution in as thin a layer as possible. However, despite the short duration of crystal solution spreading (within 1 min), salt crystals often formed in samples with a high salt concentration in the crystallization solution. To delay the evaporation of the crystal solution, we increased the loading volume to 80 μl, in which case salt from the solution was not crystallized. The crystal solution mainly surrounds the crystal within the mesh hole or lies between the mesh and the polyimide film. Once the nylon mesh is sealed inside the polyimide film, the crystal samples did not dry out after a day.

When the crystal solution was applied and spread onto a nylon mesh, most of the crystals were in the mesh pores (Supplementary Fig. S2). A diffraction pattern analysis showed that the crystals lying on the mesh are accompanied by background scattering from the nylon (Supplementary Fig. S2). This scattering was negligible for analyses of diffraction data. We evaluated the proper nylon mesh pore size for the lysozyme (30–40 μm) and glucose isomerase (<60 μm) solutions. Crystal suspensions were dispensed onto nylon mesh pores of 20, 30, 60, and 100 μm to screen the size at which the crystals were well placed on the mesh pores (Supplementary Fig. S3). Both crystal samples were relatively uniformly distributed on a nylon mesh with a 60 μm pore size (Fig. 2e,f). On the other hand, smaller crystals also can be placed on the mesh pore in the same way. However, in this case, multiple crystals can happen to be placed on single mesh pore, causing multiple crystal hits when the X-ray passes through. This can interfere with data processing. Therefore, for a small crystal sample, a nylon mesh with a pore size slightly larger than the crystal size will be required for mounting the single crystal in each mesh pore.

### Observation of the nylon mesh enclosed with polyimide after XFEL penetration

**Figure 3:**
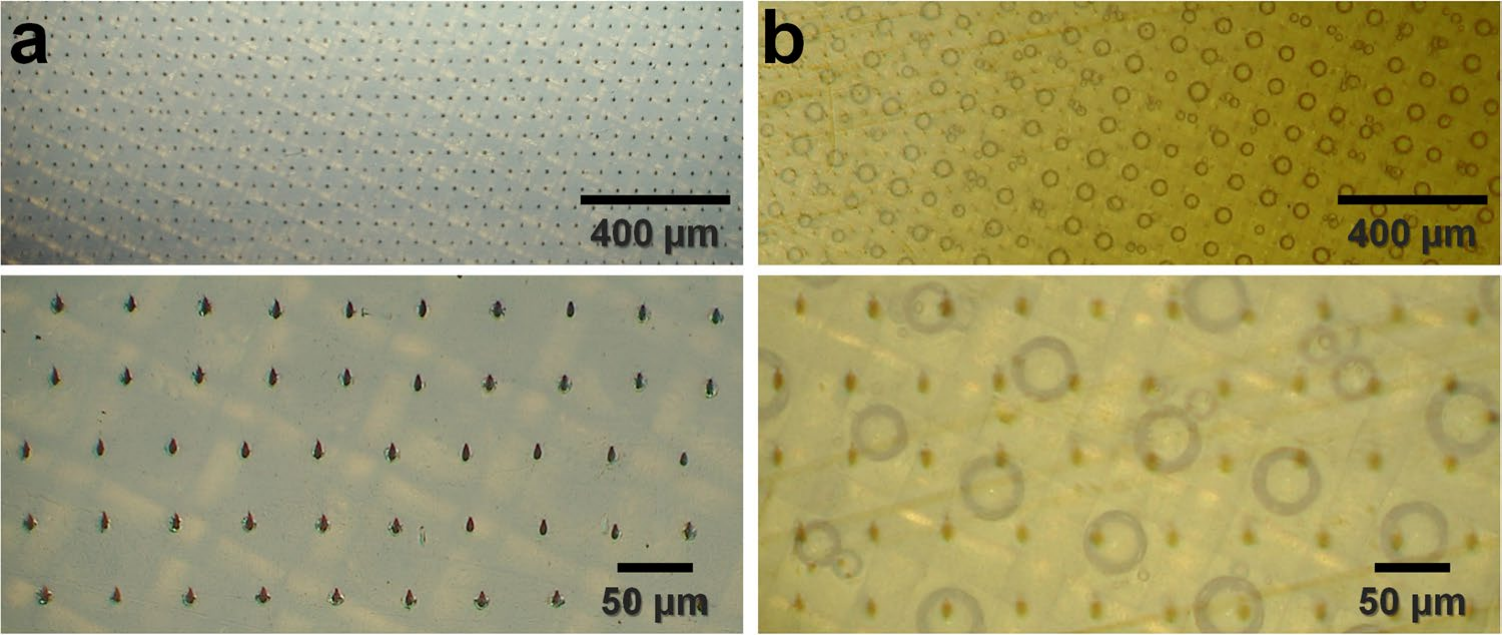
Close-up view of X-ray penetrated nylon mesh enclosed by polyimide films. (**a**) After the penetration of X-rays, a hole was formed in the outer wall of the polyimide film. There was no hole in the polyimide film where the X-ray was not transmitted. (**b**) Where the X-rays were transmitted, bubbles formed inside the polyimide film.

After XFEL data collection, we observed the nylon mesh enclosed in the polyimide film (Fig. 3). There was a hole in the surface of the polyimide film, indicating the site of focused XFEL penetration (Fig. 3a). During data collection, we set the stage to be mechanically raster-scanned at 50 μm intervals. Distance between the holes on the polyimide film, passing through the XFEL, was 50 μm (Fig. 3a), which was identical to the distance programmed in our FT-SFX system. The beam size after focusing using the KB mirror at the sample position was 4 × 8 μm (FWHM), and the size of the surface hole of polyimide generated by XFEL penetration was approximately 6 × 10 μm (Supplementary Fig. S4). We also observed that a bubble formed inside the mesh hole enclosed by polyimide films at the location where the XFEL passed (Fig. 3b). After X-ray data collection, high-temperature macromolecular crystals suddenly release gas bubble by radiation damage^38^. Similar observations have been made in cryo-EM, indicating the formation of molecular hydrogen bubbles in irradiated organic samples^38,39^. Bubbles were only observed in the mesh pores of the X-ray transmitted region (Supplementary Fig. S5), suggesting that bubbles resulted from gas emission due to radiation damage of the crystal sample or crystal solution after the XFEL passed.

### Data collection and structural analysis of FT-SFX using the nylon mesh sample holder

To demonstrate the application of the nylon mesh-based sample holder, we performed an FT-SFX experiment using lysozyme and glucose isomerase crystals as model samples. Diffraction data were collected by the raster scanning method at room temperature. Full width XFELs generate through-holes and crater radii that are larger than the focused beam size^15^. To avoid exposure of the surrounding crystal samples to physical alterations when XFEL passes the sample holder, we performed raster scanning at 50 μm intervals. The maximum available area for a nylon mesh chip, using our setup, is 20 mm × 20 mm, and it can collect a maximum of 160,000 images. We collected diffraction data at approximately 1 mm distance from the PVC frame. We used XFEL pulses with a 30 Hz repetition of a 20 fs pulse width for a photon energy of 9.7 keV with a photon flux of ~5 × 10^11^ photons per pulse.

**Figure 4:**
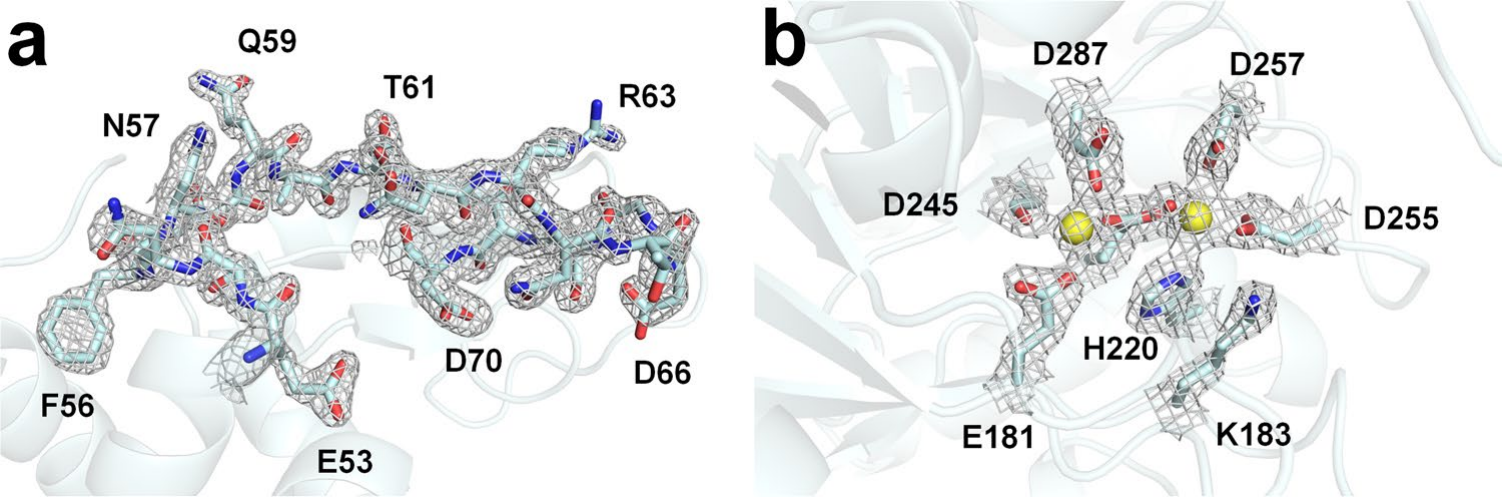
Electron density maps of lysozyme and glucose isomerase. The 2Fo-Fc electron density maps of (**a**) lysozyme (gray mesh, 1.5 σ) and (**b**) glucose isomerase (gray mesh, 1.7 σ).

For the lysozyme dataset, we obtained 133,107 images over 1.24 h. Using the Cheetah program, we obtained 118,985 images with observed diffraction with a hit rate of 89.39%. After optimization of the detector geometry, we obtained 80,177 indexed images with an indexing rate of 67.38% (Supplementary Fig. S6). Most diffraction images showed a single crystal diffraction pattern, but multiple hits also existed. Among the total indexed lysozyme images, 14.3% of images contained diffraction patterns of multiple crystals. Post-refinement was performed using *partialator* in CrystFEL, and the diffraction data for up to 1.65 Å were used. The overall signal-to-noise ratio and completeness were 6.61 and 100%, respectively. Overall *R*_split_ and CC* were 10.28 and 99.62%, respectively. The final model was refined to 1.65 Å, while *R*_work_ and *R*_free_ were 19.93% and 22.75%, respectively. The electron density map of lysozyme was very clear for the interpretation from Lys1 to Leu129, excluding the C-terminus (Fig. 4a). The lysozyme structure obtained by FT-SFX using the nylon mesh showed similarity with the lysozyme structure obtained at room temperature using a gas dynamic virtual nozzle (PDB code 4ET8)^40^, a droplet injector (5DM9)^41^, and polyacrylamide (6IG6)^9^ with an r.m.s. deviation of 0.130–0.197 Å for all Cα atoms (Supplementary Fig. S6). A structural analysis revealed clear disulfide bonds in the electron density map of lysozyme, indicating no radiation damage (Supplementary Fig. S7).

For the glucose isomerase dataset, we obtained 134,325 images over 1.24 h. Applying the same data processing used for the lysozyme dataset, we obtained 79,805 images with observed diffraction with a hit rate of 59.41%. We obtained 29,157 indexed images with an indexing rate of 36.53% (Supplementary Fig. S8). Among the total indexed glucose isomerase images, 10.8% of images contained diffraction patterns of multiple crystals. The diffraction data for glucose isomerase at up to 1.75 Å were used. The overall signal-to-noise ratio and completeness were 4.03 and 100%, respectively. Overall *R*_spirt_ and CC* were 21.63 and 98.12%, respectively. The final model was refined to 1.75 Å, while *R*_work_ and *R*_free_ were 18.18% and 20.30%, respectively. The electron density map of glucose isomerase was very clear for the interpretation from Tyr3 to Gly388 (Fig. 4b). The glucose isomerase structure obtained using the nylon mesh showed similarity with glucose isomerase at room temperature using a grease matrix (PDB code 4W4Q)^8^ with an rms deviation of 0.274 Å for all Cα atoms (Supplementary Fig. S9). To recognize and activate the substrate in glucose isomerase, the two metal binding sites in the active site must be occupied by active metals, such as Mg^2+^ or Mn^2+^ ^42,43^. For the structure of glucose isomerase using the grease matrix, the M2 site (catalytic site) was refined with Ca^2+^ ions, and there was no metal at the M1 site (substrate recognition) (Supplementary Fig. S9). As a result, this structure was close to the inactive form because there was no metal in the M1-recognizing substrate and there was a Ca^2+^ metal in the M2 site, which is irrelevant to activity. In contrast, our glucose isomerase structure using nylon mesh exhibited a clear two-metal binding mode with Mg^2+^ bound to both M1 and M2 of the active site, indicating the functional active form (Supplementary Fig. S9). This structural difference may be attributed to the method for preparing the crystal sample rather than a difference in the sample delivery method.

### Analysis of background scattering from the nylon chip enclosed by a polyimide film

**Figure 5:**
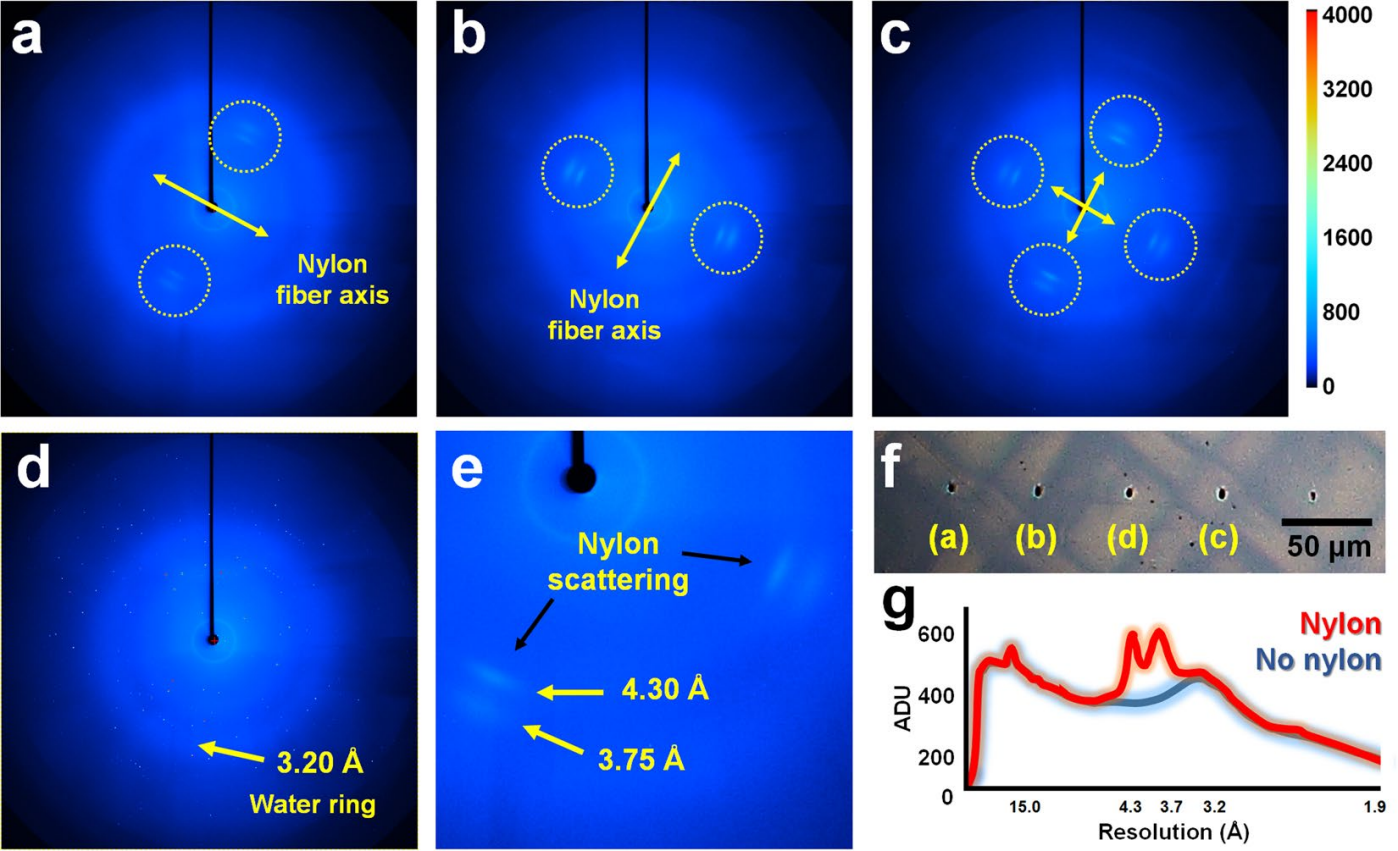
Analysis of background scattering of the nylon mesh-based sample holder. Image of nylon X-ray scattering when XFEL penetrates (**a,b**) nylon, (**c**) nylon intersections, and (**d**) mesh pores. A water ring is observed around 3.2 Å. (**e**) Close-up view of X-ray scattering from the nylon mesh. The nylon shows diffused x-ray scattering at about 3.75 Å and 4.30 Å. (**f**) Expected image of a polyimide film in which an x-ray is transmitted through a nylon, a nylon intersection, and a mesh pore. (**g**) Comparison of background scattering when X-rays penetrate nylon and mesh pores.

In addition to successfully determining crystal structures using nylon mesh enclosed by polyimide films, we further analyzed background scattering from the nylon material for more efficient applications. The raster scan was set at 50 μm intervals to avoid affecting the surrounding crystal as the X-ray passes through. On the other hand, 60 μm mesh pore refers to the size for holding the crystal stably. As a result, during raster scanning, XFEL penetrates both the mesh pore as well as the nylon mesh. We classified the diffraction images into four distinct background scattering patterns. The first background scattering pattern was observed at 3.75 and 4.30 Å in both directions relative to the beam center (Fig. 5a). This pattern indicates the X-ray scattering of nylon that appears when a typical fiber is exposed to X-rays^44^. The directions of both scattering points are parallel to the actual nylon fiber axis. The second pattern is a 90° rotated version of the first pattern, indicating that the direction of the nylon fiber is rotated 90° (Fig. 5b). The third background scattering pattern is observed in four directions with respect to the beam center (Fig. 5c). This is the location where two nylon fibers intersect in the mesh. The fourth pattern is the nylon-free image, which is transmitted through the nylon mesh pore (Fig. 5d). The expected nylon transmission positions for the four diffraction patterns are shown in Fig. 5e. The scattering intensity of nylon at 3.75 and 4.30 Å was approximately 600 AUD (Fig. 5f). In comparison to the data passing through the mesh pore, the scattering intensity in the actual nylon corresponds to approximately 200 ADU. This is a low level of scattering, with no effect on data processing. All images had background scattering of more than 400 ADU at low resolutions of below 4 Å, which corresponds to background scattering from beam stops, polyimide films, and air. In this experiment, we collected diffraction data in the atmosphere with a nylon mesh enclosed in 25 μm polyimide films. In the future, thinner polyimide or Mylar films can be used to collect data in helium environment to reduce background scattering in the low-resolution area of the diffraction data. Moreover, we used a PVC frame with a thickness of 300 μm in this experiment, whereas thickness of the crystallization solution containing the nylon mesh was about 250 μm. To reduce background scattering from the crystallization solution, the thickness between polyimide films can be reduced by using a commercially available thinner PVC plate when preparing the sample holder.

## Conclusion

We performed an FT-SFX experiment using a nylon mesh to facilitate FT-SFX. The newly developed nylon mesh-based sample holder for FT-SFX has several advantages. (1) Nylon is an X-ray transparent material that produces a weak scattering intensity. Because it does not refract X-rays, unlike silicon, it can be used without a risk of detector damage. (2) Since the negligible background scattering produced by nylon has little impact on the quality of diffraction data, rapid data acquisition by raster scanning is possible, without special sample holder alignment. (3) Since nylon meshes with a variety of pore sizes can be purchased commercially at a low cost, high selectively depending on the size and shape of the crystals is possible. Although we successfully performed FT-SFX using a nylon mesh, we can improve the quality of diffraction data by removing the X-ray scattering of nylon. We are currently developing an image processing method that allows the XFEL to pass through only the pores of the nylon mesh when performing FT-SFX.

## Methods

### Sample preparation

Lysozyme powder from chicken egg white was purchased from Hampton Research (Cat No. HR7-110; Aliso Viejo, CA, USA) and dissolved in 10 mM Tris-HCl, pH 8.0, to a final lysozyme concentration of 50 mg/ml. Lysozyme was crystallized using a solution containing 0.1 M sodium acetate, pH 4.0, 6% (w/v) polyethylene glycol 8000 and 200 M NaCl. Glucose isomerase from *Streptomyces rubiginosus* was purchased from Hampton Research (Cat No. HR7-102). Glucose isomerase (33 mg/ml) was stored in a solution containing 6 mM Tris-HCl, pH, 7.0, 0.91 M ammonium sulfate, and 1 mM magnesium sulfate, and yielded glucose isomerase crystals of various sizes. Crystals in the glucose isomerase solution were used directly, without separate crystallization experiments. The crystal sizes of lysozyme and glucose isomerase used in the experiments were 20–30 μm and <60 μm, respectively.

### Fabrication of the nylon mesh-based sample holder

A nylon mesh with a mesh pore size of 60 μm was purchased from Merck (Cat. No. NY6004700; Burlington, MA, USA). The 25 μm polyimide film was purchased from Covalue Youngjin Co. (Daegu, Republic of Korea). The polyimide film was fixed to a PVC (Crenjoy, Seoul, Republic of Korea) frame using double-sided adhesive polyimide tape (Daehyunst, Hwasung, Republic of Korea). The nylon mesh was placed on a polyimide film, and an 80 μl volume of crystal solution was loaded evenly using a pipette. Thereafter, the polyimide film was immediately covered and the two polyimide films were enclosed using a double-sided adhesive polyimide film.

### Data collection

The FT-SFX experiment using the nylon mesh with X-ray pulses was performed at the NCI (Nano Crystallography and Coherence Imaging) experimental hutch at PAL-XFEL^45,46^. The X-ray energy was 9.7 keV (1.2782 Å) with a photon flux of ~5 × 10^11^ photons per pulse within a 20 fs duration. The X-ray pulse was focused to 4 (horizontal) × 8 (vertical) μm^2^ (FWHM) using a Kirkpatrick-Baez mirror^47^. The data were collected in atmosphere at room temperature and recoded using the MX225-HS (Rayonix, LLC, Evanston, IL, USA) detector with a 4 × 4 binning mode (pixel size: 156 μm × 156 μm). The motion stage for FT-SFX was designed to allow raster scanning of up to maximum 60 Hz beam that is provided by PAL-XFEL, and was custom-built by SmartAct. We used piezo SLLV42 (SmartAct) and SLL12 (SmartAct) actuator for translation in the horizontal and vertical directions, respectively. During the raster scanning, the acrylic support containing the nylon mesh sample holder was translated within 18 mm in both vertical and horizontal directions using a piezo linear stage in the sample chamber. This scanning stage is motioned by remote control system, without synchronization for the arrival FEL pulses. A raster scan for sample holder was performed from the top to the bottom direction. The sample holder containing the crystals was scanned at 50 μm intervals from left to right, and then moved to the bottom by 50 μm. Next scan was performed from right to left at 50 μm intervals, and then moved to the bottom by 50 μm. These raster scanning motions were repeatedly performed to collect full dataset. The velocity of the sample holder mounted in the motion stage was 1.5 mm/s for both horizontal and vertical directions. Diffraction data through raster scanning was performed in ambient pressure at room temperature.

### Data processing and structure determination

The diffraction pattern was monitored using OnDA^48^. The hit images were filtered using Cheetah^49^. The diffraction images were indexed, integrated, merged, and post-refined using CrystFEL^50^. The phasing of the lysozyme was obtained by molecule replacement using the Phaser-MR in PHENIX^51^ with lysozyme (PDB code 6IG6)^9^ as the search model. The phasing of glucose isomerase was obtained by molecule replacement using the Phaser-MR in PHENIX^51^ with glucose isomerase (PDB code 5Y4J)^43^ as the search model. Model building and refinement were performed using Coot^52^ and Phenix.refinement in PHENIX^51^, respectively. The geometry of the final model was validated using MolProbity^53^. Figures were generated using PyMOL (available at https://pymol.org/). The data collection and structural refinement statistics are shown in Table 1.

**Table 1:** Data collection and refinement statistics.

Data collection	Lysozyme	Glucose isomerase
Energy (eV)	9700	9700
Photons/pulse	~5 × 10^11^	~5 × 10^11^
Pulse width^a^	20 fs	20 fs
Space group	P4_3_2_1_2	I222
**Cell dimensions**		
*a*, *b*, *c* (Å)	78.22, 78.22, 37.76	93.05, 99.00, 101.92
No. collected diffraction images	133107	134325
No. of hits	118985	79805
No. of indexed images	80177	29157
No. of unique reflections	29127	47861
Resolution (Å)	80.0–1.65 (1.71–1.65)	71.94–1.75 (1.81–1.75)
Completeness	100.0 (100.0)	100.0 (100.0)
Redundancy	4660.8 (962.2)	356.8 (125.2)
*I/σ(I)*	6.61 (1.36)	4.03 (1.45)
*R*_split_^b^	10.28 (78.73)	21.63 (64.71)
CC*(%)	99.62 (73.90)	98.12 (94.15)
Wilson B factor (Å^2^)	50.38	43.81
**Refinement statistics**		
Resolution (Å)	78.22–1.65	71.01–1.75
R_factor_/R_free_ (%)^c^	19.93/22.75	18.18/20.30
**B-factor (Averaged)**		
Protein	43.40	40.06
Metal	41.55	30.96
Water	45.70	43.24
**R.m.s. deviations**		
Bond lengths (Å)	0.010	0.010
Bond angles (°)	1.071	1.078
**Ramachandran plot (%)**		
favored	98.43	96.9
allowed	1.57	2.8
outlier		0.3

Highest resolution shell is shown in parentheses.

^a^Electron bunch length.

^b^$${R}_{split}=(1/\sqrt{2})\cdot \frac{{\sum }_{hkl}|{I}_{hkl}^{even}-{I}_{hkl}^{odd}|}{\frac{1}{2}|{I}_{hkl}^{even}-{I}_{hkl}^{odd}|}$$.

^c^*R*_work_ = Σ||*F*_obs_| − |*F*_calc_||/Σ|*F*_obs_|, where *F*_obs_ and *F*_calc_ are the observed and calculated structure-factor amplitudes respectively. R_free_ was calculated as R_work_ using a randomly selected subset (10%) of unique reflections not used for structure refinement.

### Accession codes

The coordinates and structure factors have been deposited in the Protein Data Bank under the accession code 6IRJ (lysozyme) and 6IRK (Glucose Isomerase). Diffraction images have been deposited to CXIDB under ID 89 (Lysozyme) and 90 (Glucose Isomerase).

## Supplementary information

Supplementary Data

## References

[1] Schlichting I (2015). Serial femtosecond crystallography: the first five years. IUCrJ.

[2] Chavas LM, Gumprecht L, Chapman HN (2015). Possibilities for serial femtosecond crystallography sample delivery at future light sources. Struct Dyn.

[3] DePonte D P, Weierstall U, Schmidt K, Warner J, Starodub D, Spence J C H, Doak R B (2008). Gas dynamic virtual nozzle for generation of microscopic droplet streams. Journal of Physics D: Applied Physics.

[4] Sierra RG (2012). Nanoflow electrospinning serial femtosecond crystallography. Acta Crystallogr D Biol Crystallogr.

[5] Weierstall U (2014). Lipidic cubic phase injector facilitates membrane protein serial femtosecond crystallography. Nat Commun.

[6] Roessler CG (2016). Acoustic Injectors for Drop-On-Demand Serial Femtosecond Crystallography. Structure.

[7] Kovacsova G (2017). Viscous hydrophilic injection matrices for serial crystallography. IUCrJ.

[8] Sugahara M (2015). Grease matrix as a versatile carrier of proteins for serial crystallography. Nat Methods.

[9] Park J (2019). Polyacrylamide injection matrix for serial femtosecond crystallography. Sci Rep.

[10] Nam Ki (2019). Sample Delivery Media for Serial Crystallography. International Journal of Molecular Sciences.

[11] Hunter MS (2014). Fixed-target protein serial microcrystallography with an x-ray free electron laser. Sci Rep.

[12] Mueller C (2015). Fixed target matrix for femtosecond time-resolved and *in situ* serial micro-crystallography. Struct Dyn.

[13] Oghbaey S (2016). Fixed target combined with spectral mapping: approaching 100% hit rates for serial crystallography. Acta Crystallogr D Struct Biol.

[14] Roedig P (2017). High-speed fixed-target serial virus crystallography. Nat Methods.

[15] Doak RB (2018). Crystallography on a chip - without the chip: sheet-on-sheet sandwich. Acta Crystallogr D Struct Biol.

[16] Suga M (2015). Native structure of photosystem II at 1.95 A resolution viewed by femtosecond X-ray pulses. Nature.

[17] Cohen AE (2014). Goniometer-based femtosecond crystallography with X-ray free electron lasers. Proc Natl Acad Sci USA.

[18] Pedrini B (2014). 7 A resolution in protein two-dimensional-crystal X-ray diffraction at Linac Coherent Light Source. Philos Trans R Soc Lond B Biol Sci.

[19] Baxter EL (2016). High-density grids for efficient data collection from multiple crystals. Acta Crystallogr D Struct Biol.

[20] Keedy, D. A. *et al*. Mapping the conformational landscape of a dynamic enzyme by multitemperature and XFEL crystallography. *Elife* **4** (2015). 10.7554/eLife.07574 PMC4721965 26422513

[21] Lyubimov AY (2015). Capture and X-ray diffraction studies of protein microcrystals in a microfluidic trap array. Acta Crystallogr D Biol Crystallogr.

[22] Chreifi G (2016). Crystal structure of the pristine peroxidase ferryl center and its relevance to proton-coupled electron transfer. Proc Natl Acad Sci USA.

[23] Feld GK (2015). Low-Z polymer sample supports for fixed-target serial femtosecond X-ray crystallography. J Appl Crystallogr.

[24] Frank M (2014). Femtosecond X-ray diffraction from two-dimensional protein crystals. IUCrJ.

[25] Halsted TP (2018). An unprecedented dioxygen species revealed by serial femtosecond rotation crystallography in copper nitrite reductase. IUCrJ.

[26] Murray TD (2015). A high-transparency, micro-patternable chip for X-ray diffraction analysis of microcrystals under native growth conditions. Acta Crystallogr D Biol Crystallogr.

[27] Barnes CO (2018). Structural characterization of a highly-potent V3-glycan broadly neutralizing antibody bound to natively-glycosylated HIV-1 envelope. Nat Commun.

[28] Martiel I, Muller-Werkmeister HM, Cohen AE (2019). Strategies for sample delivery for femtosecond crystallography. Acta Crystallogr D Struct Biol.

[29] Roedig P (2016). Room-temperature macromolecular crystallography using a micro-patterned silicon chip with minimal background scattering. J Appl Crystallogr.

[30] Sanchez-Weatherby J (2009). Improving diffraction by humidity control: a novel device compatible with X-ray beamlines. Acta Crystallogr D Biol Crystallogr.

[31] Roedig P (2015). A micro-patterned silicon chip as sample holder for macromolecular crystallography experiments with minimal background scattering. Sci Rep.

[32] Weinert T (2017). Serial millisecond crystallography for routine room-temperature structure determination at synchrotrons. Nat Commun.

[33] Dasgupta S, Hammond WB, Goddard WA (1996). Crystal structures and properties of nylon polymers from theory. J Am Chem Soc.

[34] Teng TY (1990). Mounting of Crystals for Macromolecular Crystallography in a Freestanding Thin-Film. Journal of Applied Crystallography.

[35] Bhuvanesh NSP, Reibenspies JH (2003). A novel approach to micro-sample X-ray powder diffraction using nylon loops. Journal of Applied Crystallography.

[36] Mathews II (2017). The Conformational Flexibility of the Acyltransferase from the Disorazole Polyketide Synthase Is Revealed by an X-ray Free-Electron Laser Using a Room-Temperature Sample Delivery Method for Serial Crystallography. Biochemistry.

[37] Lobley CM (2016). A generic protocol for protein crystal dehydration using the HC1b humidity controller. Acta Crystallogr D Struct Biol.

[38] Meents A, Gutmann S, Wagner A, Schulze-Briese C (2010). Origin and temperature dependence of radiation damage in biological samples at cryogenic temperatures. P Natl Acad Sci USA.

[39] Leapman RD, Sun S (1995). Cryo-electron energy loss spectroscopy: observations on vitrified hydrated specimens and radiation damage. Ultramicroscopy.

[40] Boutet S (2012). High-resolution protein structure determination by serial femtosecond crystallography. Science.

[41] Mafune F (2016). Microcrystal delivery by pulsed liquid droplet for serial femtosecond crystallography. Acta Crystallogr D Struct Biol.

[42] Bae JE, Hwang KY, Nam KH (2018). Structural analysis of substrate recognition by glucose isomerase in Mn(2+) binding mode at M2 site in S. rubiginosus. Biochem Biophys Res Commun.

[43] Bae JE, Kim IJ, Nam KH (2017). Crystal structure of glucose isomerase in complex with xylitol inhibitor in one metal binding mode. Biochem Biophys Res Commun.

[44] Matyi RJ, Crist B (1978). Small-Angle X-Ray-Scattering by Nylon-6. J Polym Sci Pol Phys.

[45] Park J, Kim S, Nam KH, Kim B, Ko IS (2016). Current status of the CXI beamline at the PAL-XFEL. J Korean Phys Soc.

[46] Kang HS (2017). Hard X-ray free-electron laser with femtosecond-scale timing jitter. Nat Photonics.

[47] Kim J (2018). Focusing X-ray free-electron laser pulses using Kirkpatrick-Baez mirrors at the NCI hutch of the PAL-XFEL. J Synchrotron Radiat.

[48] Mariani V (2016). OnDA: online data analysis and feedback for serial X-ray imaging. J Appl Crystallogr.

[49] Barty A (2014). Cheetah: software for high-throughput reduction and analysis of serial femtosecond X-ray diffraction data. J Appl Crystallogr.

[50] White TA (2012). CrystFEL: a software suite for snapshot serial crystallography. J Appl Crystallogr.

[51] Adams PD (2010). PHENIX: a comprehensive Python-based system for macromolecular structure solution. Acta Crystallogr D Biol Crystallogr.

[52] Emsley P, Cowtan K (2004). Coot: model-building tools for molecular graphics. Acta Crystallogr D Biol Crystallogr.

[53] Chen VB (2010). MolProbity: all-atom structure validation for macromolecular crystallography. Acta Crystallogr D.

