# Supplementary material for: Nylon mesh-based sample holder for fixed-target serial femtosecond crystallography
Source: Sci Rep. 2019 May 6;9:6971. doi: 10.1038/s41598-019-43485-z (PMC6502819; doi:10.1038/s41598-019-43485-z)
Supplement: Supplementary file 1 — Supplementary Data [file 41598_2019_43485_MOESM1_ESM.docx]

**Supplementary Data**

**Nylon mesh based sample holder for fixed target serial femtosecond crystallography**

Donghyeon Lee^1,7^, Sangwon Baek^2,7^, Jaehyun Park^3,7^, Keondo Lee^1^, Jangwoo Kim^3^, Sang Jae Lee^3^, Wan Kyun Chung^1^, Jong-Lam Lee^2^, Yunje Cho^4^, Ki Hyun Nam^5,6,*^

^1^ Department of Mechanical Engineering, POSTECH, Pohang 37673, Republic of Korea.

^2^ Department of Materials Science and Engineering, POSTECH, Pohang 37673, Republic of Korea.

^3^ Pohang Accelerator Laboratory, Pohang 37673, Republic of Korea.

^4^ Department of Life Science, POSTECH, Pohang 37673, Republic of Korea.

^5^ Division of Biotechnology, Korea University, Seoul 02841, Republic of Korea.

^6^ Institute of Life Science and Natural Resources, Korea University, Seoul 02841, Republic of Korea.

^7^ These authors contributed equally to the work.

*Corresponding author.: Division of Biotechnology, Korea University, Seoul, Republic of Korea. E-mail: structures@korea.ac.kr (K.H.N.)


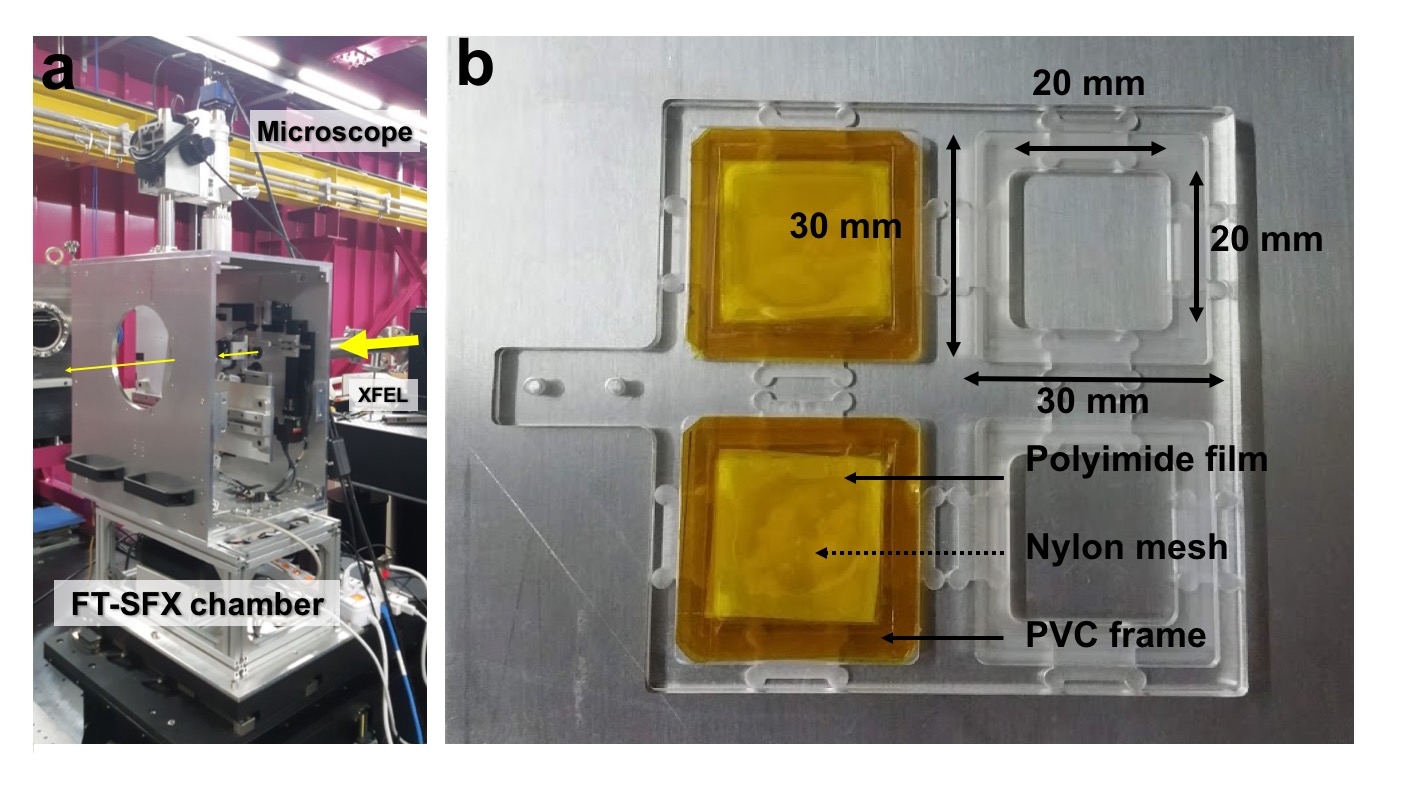


**Supplementary Figure S1. Fixed-target serial femtosecond crystallography (FT-SFX) system using nylon mesh enclosed by a polyimide film.** (a) FT-SFX chamber. (b) Photo of a sample holder mounting plate made with acryl, which is installed into the motion stage.


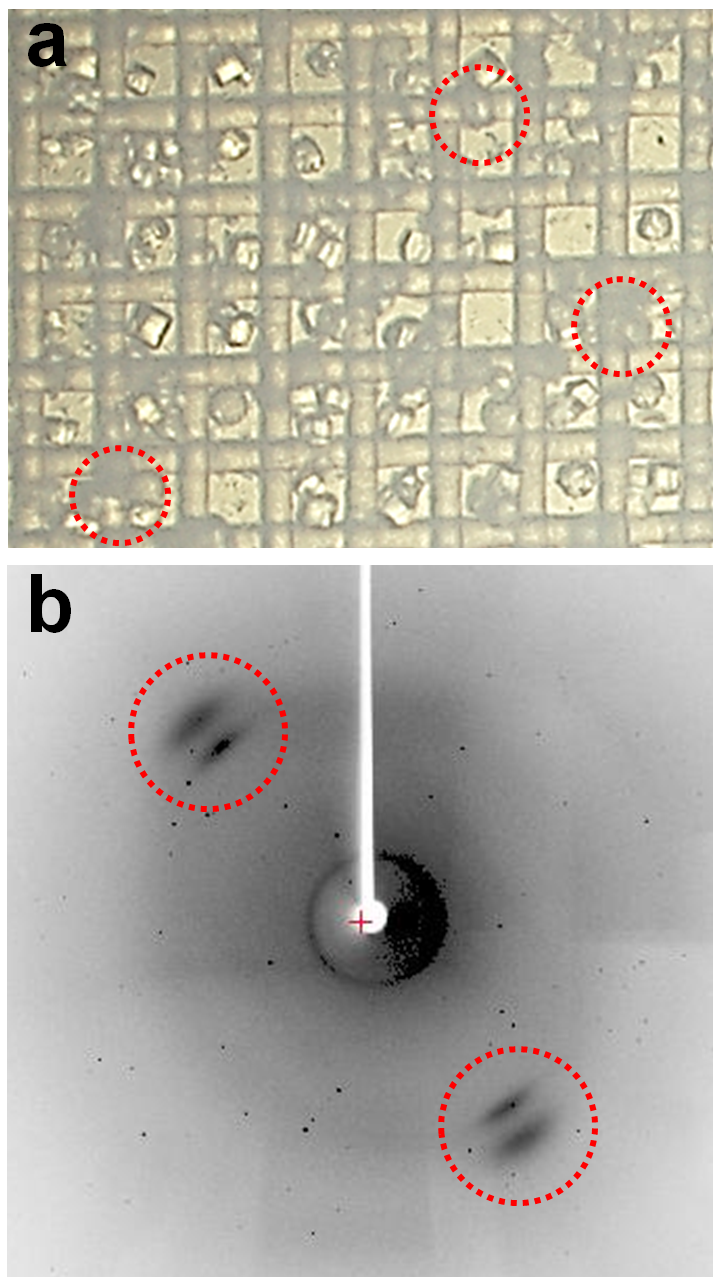


**Supplementary Figure 2. Crystal distribution on the nylon mesh sample holder.** (a) Most of the lysozyme crystal samples are located on the pore of the nylon mesh, whereas some of the crystal samples are on the nylon mesh (dashed red circle). (b) Crystal samples on the nylon mesh generate not only Bragg peaks from the crystal sample but also background scattering from the nylon mesh (dashed red circle).


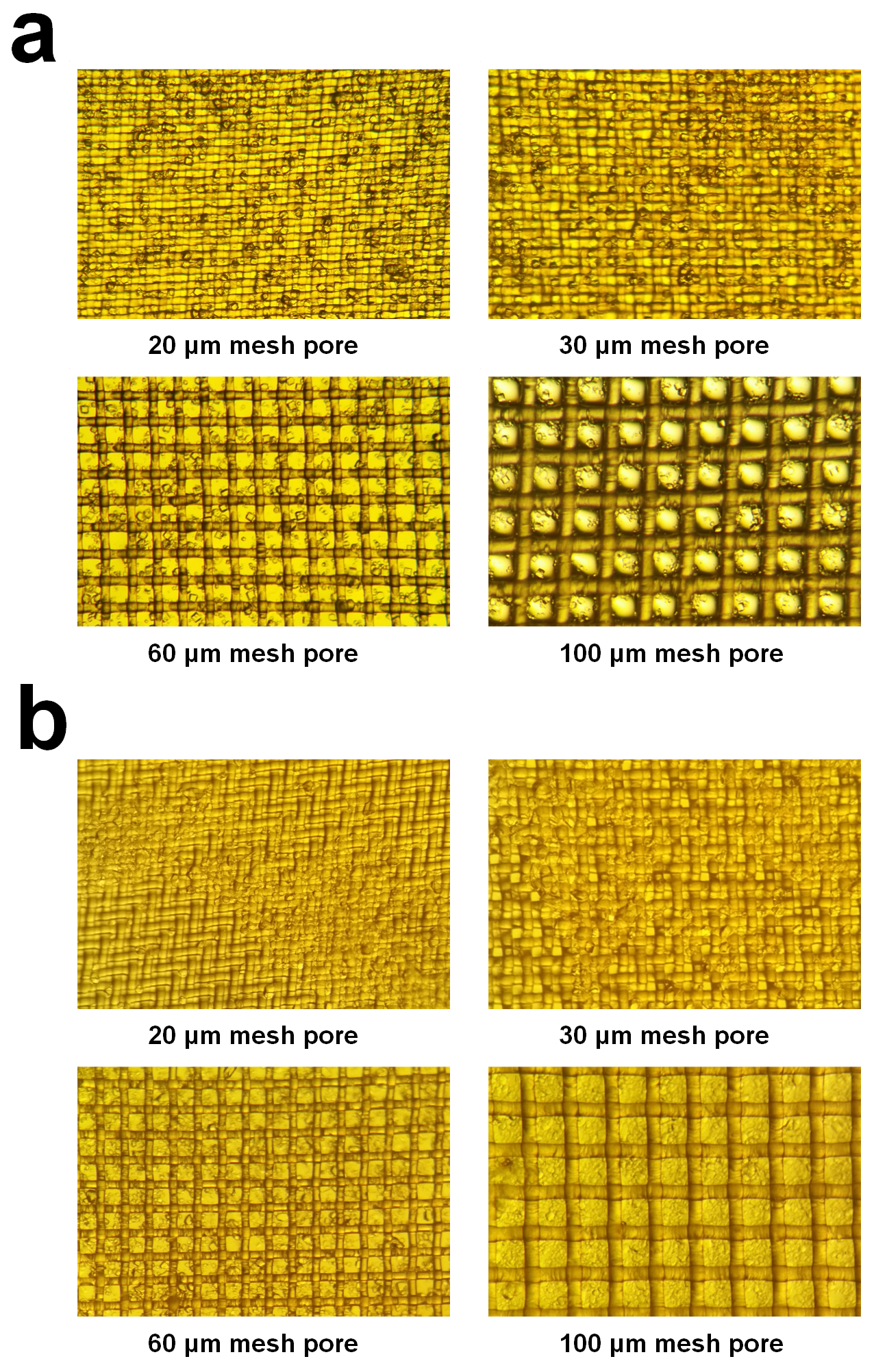


**Supplementary Figure S3. Photographs of a (a) lysozyme and (b) glucose isomerase crystal sample loaded onto a nylon mesh with 20, 30, 60, and 100 μm pore size.**


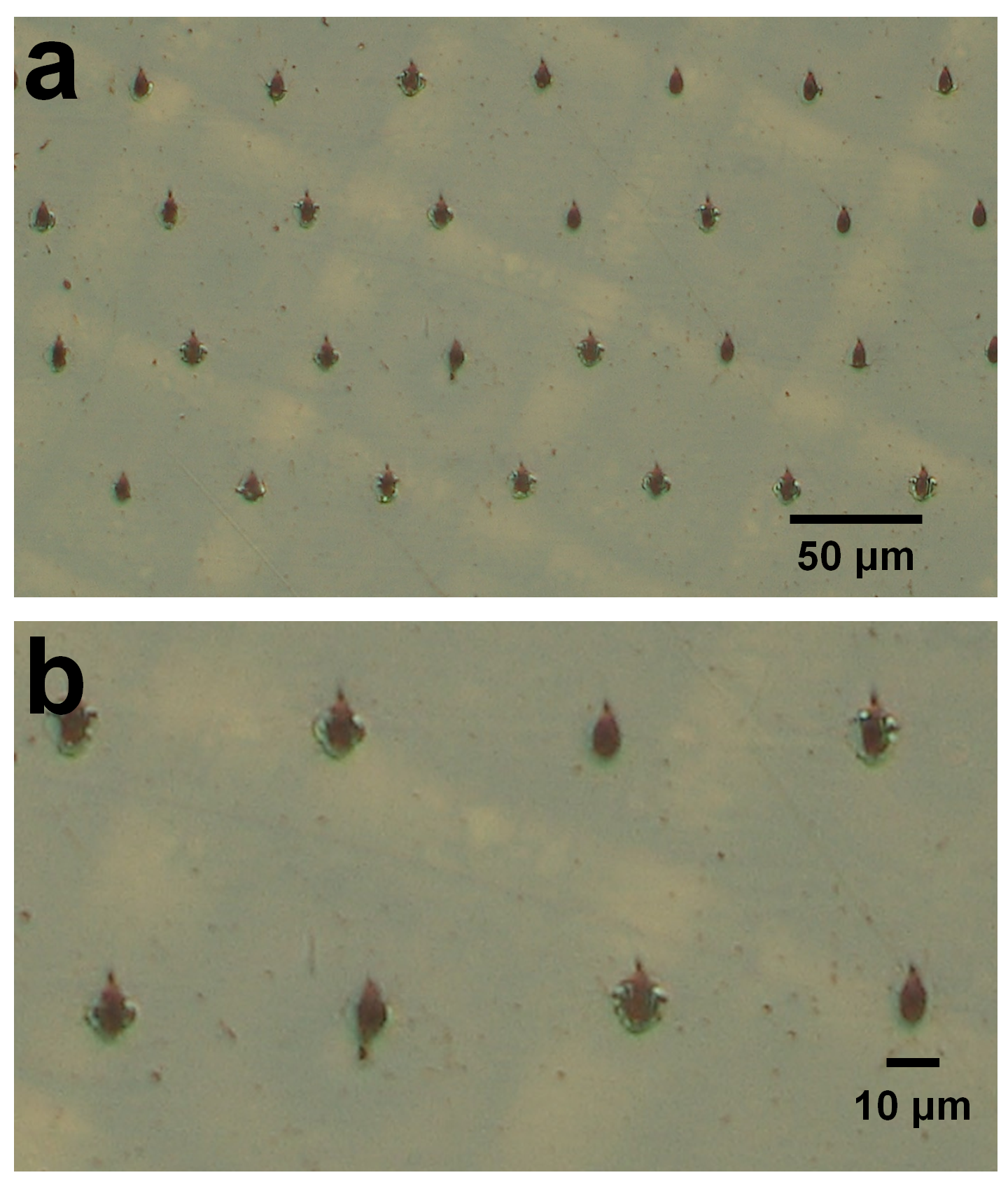


**Supplementary Figure S4. Close-up view of the X-ray penetrated surface of the polyimide film.** (a) The hole generated by XFEL penetration had an interval of 50 μm. (b) Close-up view of the X-ray-penetrated hole. The beam size at the sample position is 4 μm (horizontal) × 8 μm (vertical) (FWHM), and the spot where XFEL penetrated is larger than the focused beam size.


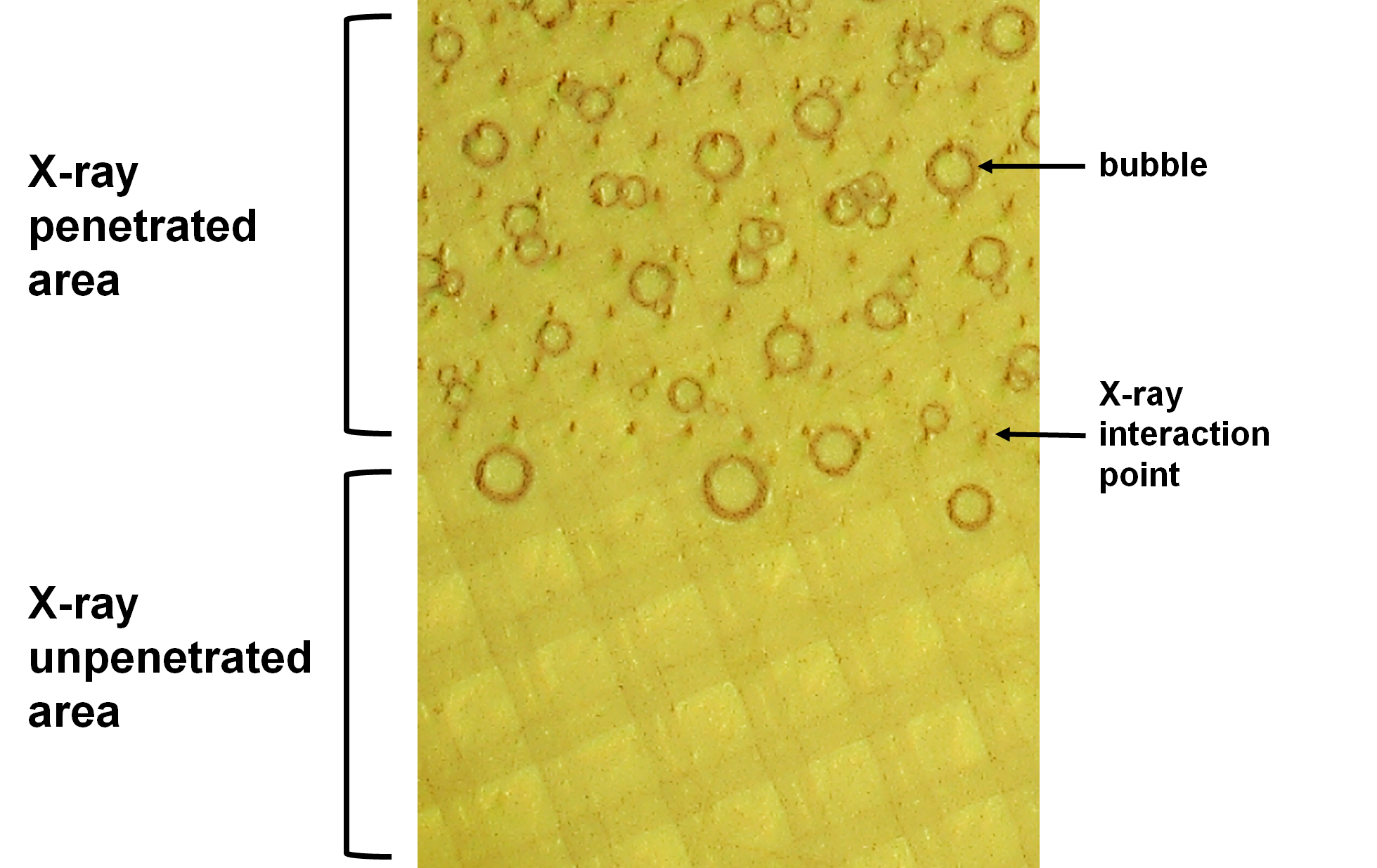


**Supplementary Figure S5. Close-up view of the bubble in the nylon mesh enclosed by the polyimide film after X-ray penetration.** Bubbles are only observed on the X-ray-penetrated area. We consider that the radiation-damaged protein crystal or solution released the gas. Bubbles are often generated in places where X-rays are not transmitted, regarded as areas where the solution is not evenly distributed during crystal distribution.

**
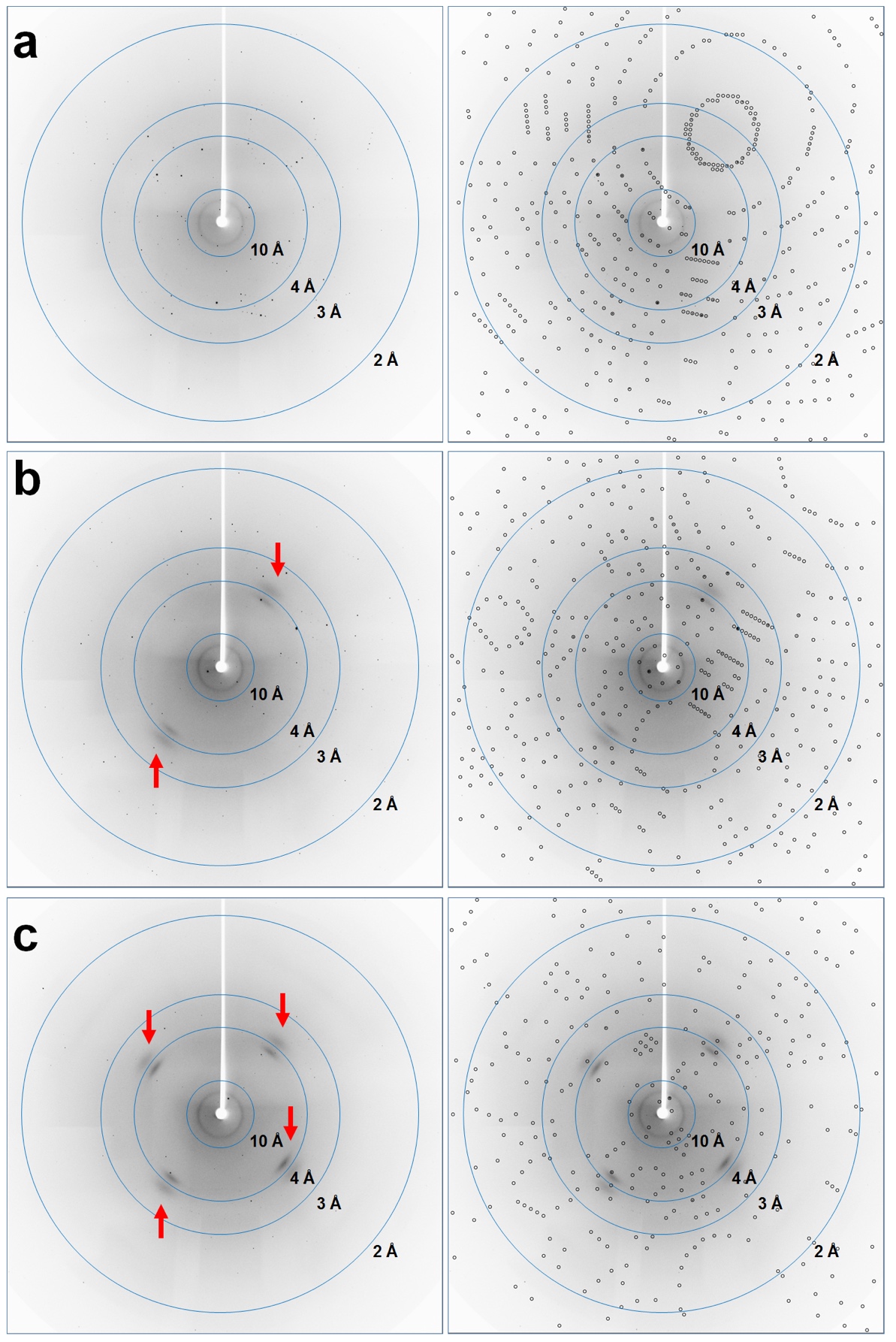
**

**Supplementary Figure S6.** Diffraction pattern (left) and indexed imaged (right) of lysozyme by FT-SFX using the nylon mesh enclosed by the polyimide film. Scattering analysis shows that XFEL penetrates (a) a mesh pore without nylon scattering, (b) a nylon mesh, and (c) the intersection of nylon mesh. Nylon scattering is indicated by red arrows. Nylon scattering does not significantly influence the indexing of the diffraction pattern by CrystFEL.

**
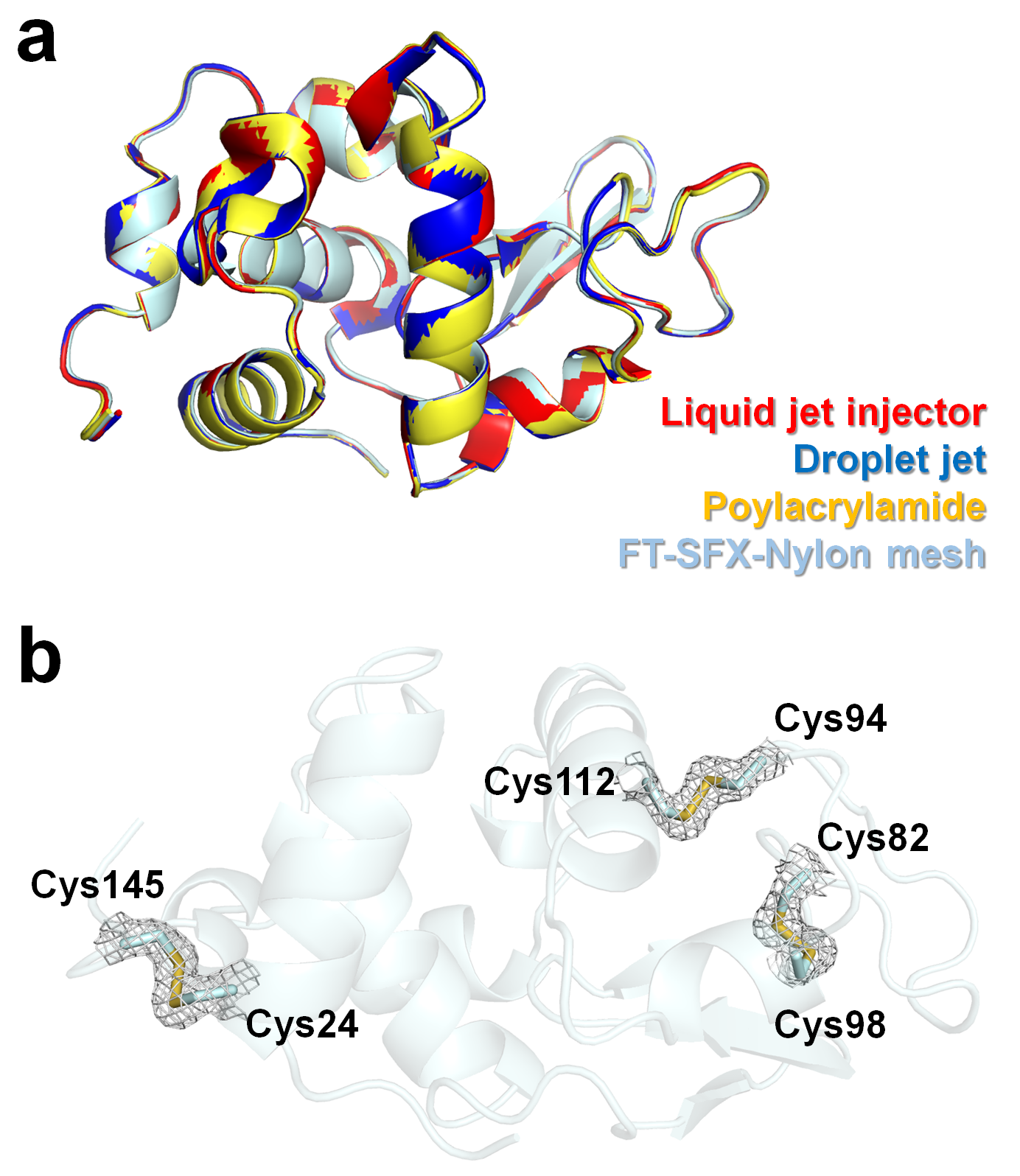
**

**Supplementary Figure S7**. (a) Superimposition of the crystal structure of lysozyme by FT-SFX using the nylon mesh enclosed by a polyimide film (cyan) and lysozyme delivered from a liquid jet injector (PDB code: 4ET8, red, r.m.s. deviation: 0.130 Å), Droplet (5DM9, blue, 0.166 Å), and polyacrylamide (6IG6, yellow, 0.197 Å). (b) 2Fo-Fc electron density map (grey, counted 1.5 σ) of disulfide bonds in lysozyme by FT-SFX using a nylon mesh enclosed by a polyimide film.


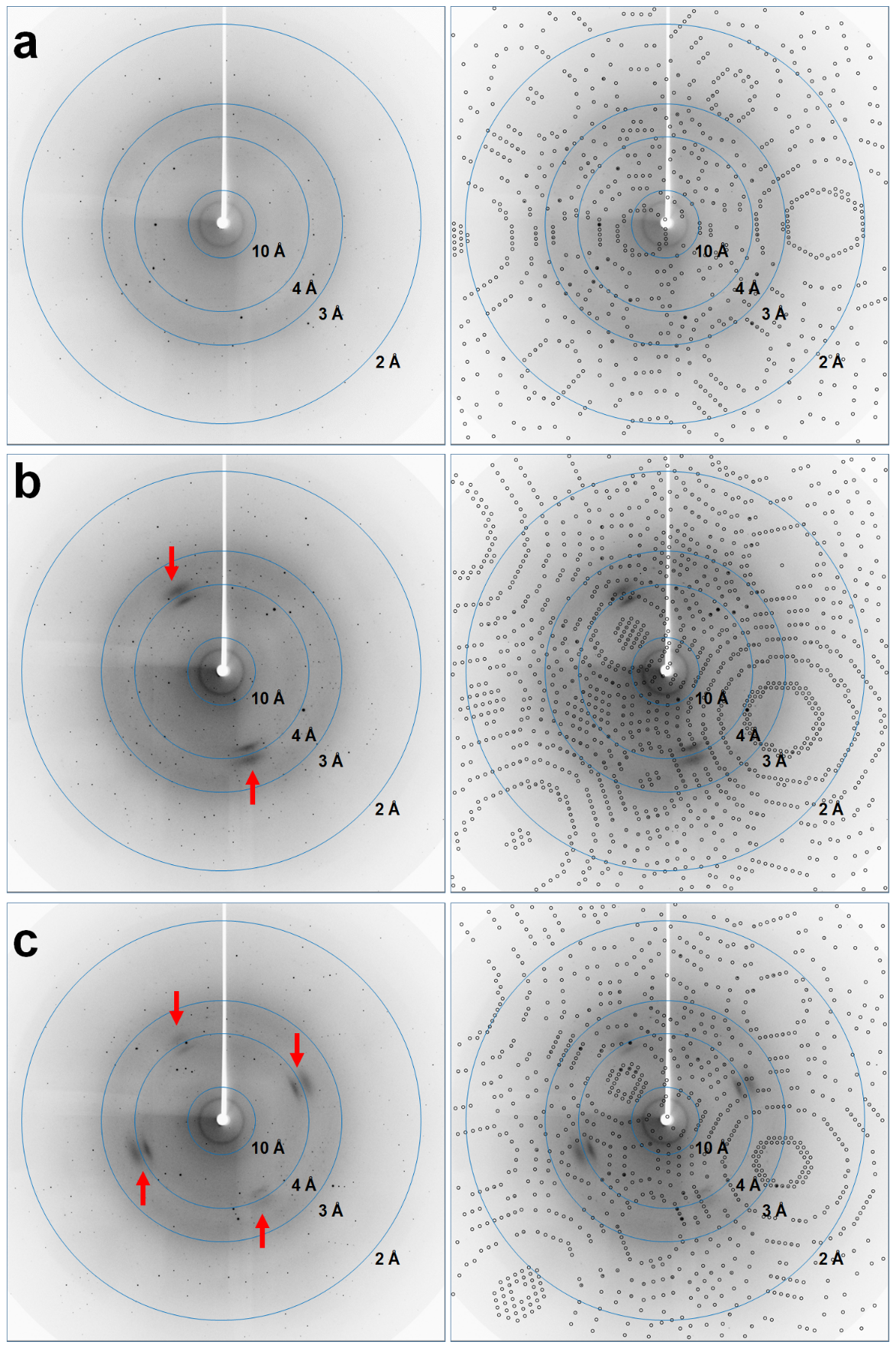


**Supplementary Figure S8.** Diffraction pattern (left) and indexed imaged (right) of glucose isomerase by FT-SFX using the nylon mesh enclosed by a polyimide film. Scattering analysis shows that XFEL penetrates (a) the mesh pore without nylon scattering, (b) a nylon mesh, and (c) the intersection of nylon mesh. Nylon scattering is indicated by red arrows. Nylon scattering is negligible for the indexing of the diffraction pattern by CrystFEL.


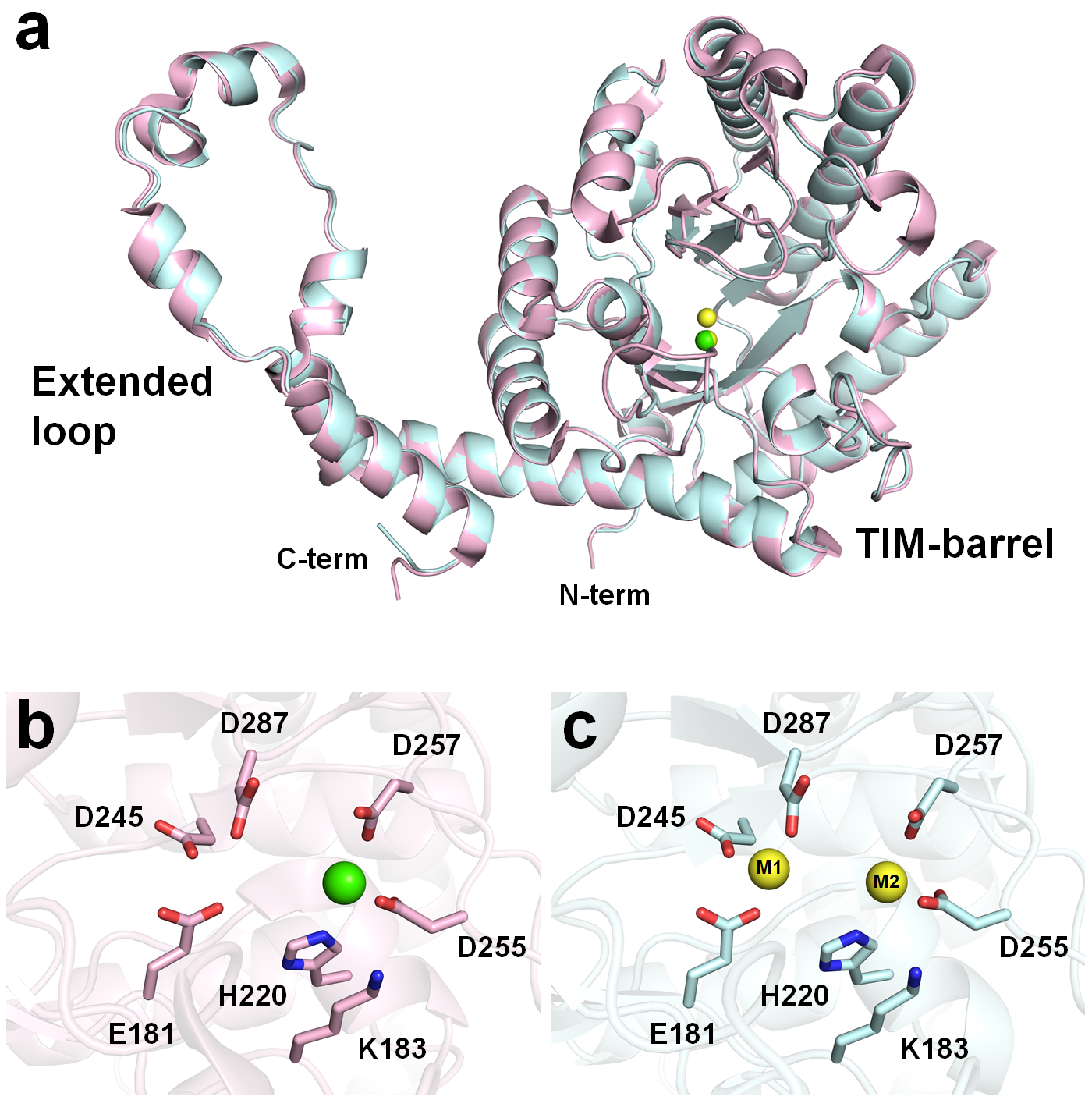


**Supporting Figure S9.** (a) Superimposition of the crystal structure of glucose isomerase by FT-SFX using nylon mesh enclosed by a polyimide film (cyan) and glucose isomerase delivered from the grease matrix (PDB code: 4W4Q, pink) with an r.m.s. deviation of 0.274 Å. (b) The metal binding site of the active site of glucose isomerase using a grease matrix. The Ca ion existed at the M2 site but was not present at the M1 site (orange dotted circle). (c) The metal binding site of the active site of glucose isomerase by FT-SFX using the nylon mesh. Mg ions are bound to the M1 and M2 sites, indicating the active configuration for glucose isomerase activity.
